# Neutron sub-micrometre tomography from scattering data

**DOI:** 10.1107/S2052252520010295

**Published:** 2020-08-20

**Authors:** B. Heacock, D. Sarenac, D. G. Cory, M. G. Huber, J. P. W. MacLean, H. Miao, H. Wen, D. A. Pushin

**Affiliations:** a Department of Physics, North Carolina State University, Raleigh, NC 27695, USA; b Triangle Universities Nuclear Laboratory, Durham, NC 27708, USA; c Institute for Quantum Computing, University of Waterloo, Waterloo, Ontario, Canada N2L3G1; d Department of Physics, University of Waterloo, Waterloo, Ontario, Canada N2L3G1; e Department of Chemistry, University of Waterloo, Waterloo, Ontario, Canada N2L3G1; f Perimeter Institute for Theoretical Physics, Waterloo, Ontario, Canada N2L2Y5; g Canadian Institute for Advanced Research, Toronto, Ontario, Canada M5G 1Z8; h National Institute of Standards and Technology, Gaithersburg, MD 20899, USA; i Biophysics and Biochemistry Center, National Heart, Lung and Blood Institute, National Institutes of Health, Bethesda, Maryland, USA

**Keywords:** computed tomography, nanoscience, nanostructures, neutron scattering, neutron diffraction, phase retrieval

## Abstract

Neutrons are valuable probes for various material samples across many areas of research. Neutron imaging typically has a spatial resolution of larger than 20 µm, whereas neutron scattering is sensitive to smaller features but does not provide a real-space image of the sample. A computed-tomography technique is demonstrated that uses neutron-scattering data to generate an image of a periodic sample with a spatial resolution of ∼300 nm. The achieved resolution is over an order of magnitude smaller than the resolution of other forms of neutron tomography. This method consists of measuring neutron diffraction using a double-crystal diffractometer as a function of sample rotation and then using a phase-retrieval algorithm followed by tomographic reconstruction to generate a map of the sample’s scattering-length density. Topological features found in the reconstructions are confirmed with scanning electron micrographs. This technique should be applicable to any sample that generates clear neutron-diffraction patterns, including nanofabricated samples, biological membranes and magnetic materials, such as skyrmion lattices.

## Abstract 2

Neutron-scattering data are passed into a phase-recovered computed-tomography algorithm, producing real-space reconstructions of periodic samples. The spatial resolution of the reconstructions is much smaller than what is achievable using other forms of neutron computed tomography.

## 1. Introduction   

Neutron imaging and scattering provide a unique probe for a wide variety of materials, motivating the construction of a growing number of neutron-imaging and -scattering user facilities (Chen & Wang, 2016 ▸; Garoby, 2017 ▸). Cold neutrons have wavelengths similar to X-rays, but whereas X-rays interact strongly with high-*Z* atoms, neutrons tend to scatter off of materials with a high hydrogen content or other light nuclei. While many imaging and scattering techniques, such as radiography and computed tomography (CT), are shared between X-rays and neutrons, the two radiation sources provide complementary information (Strobl *et al.*, 2009 ▸) and have even recently been combined in a single apparatus (LaManna *et al.*, 2017 ▸; Chiang *et al.*, 2017 ▸).

For typical applications, neutron imaging is sensitive to sample features larger than ∼20 µm. The spatial resolution of neutron imaging is limited by the resolution of neutron position-sensitive detectors (PSDs), though a range of techniques can push neutron-imaging resolution down to a few micrometres (Hussey *et al.*, 2017 ▸; Harti *et al.*, 2017 ▸; Williams *et al.*, 2012 ▸; Strobl *et al.*, 2009 ▸), and Fourier-space imaging techniques can alleviate the need for PSDs all together (Pushin *et al.*, 2006 ▸). In particular, neutron CT is a very useful form of neutron imaging and has been demonstrated with radiographic, phase-contrast, differential phase-contrast and dark-field signals (Dubus *et al.*, 2005 ▸; Strobl *et al.*, 2004 ▸, 2008 ▸, 2009 ▸).

Conversely, neutron scattering is sensitive to much smaller sample features but does not traditionally provide an image of the sample. Because neutron scattering is a far-field measurement, the role of resolution is reversed when compared with neutron imaging. Higher-resolution scattering data provide information about larger-scale features in real space. We used neutron-scattering data with a phase-retrieval algorithm to perform neutron CT on a periodic sample. Our method consists of measuring neutron diffraction as a function of sample rotation and using a phase-retrieval algorithm to recover the phase in position space. The recovered phase as a function of sample rotation is then tomographically reconstructed, providing a two-dimensional density map of the sample with a spatial resolution that is ∼300 nm and independent of the pixel size of neutron PSDs.

Neutron diffraction from the samples was measured using a double-crystal diffractometer, similar to typical ultra small angle neutron-scattering (USANS) techniques. In this first demonstration, we imaged silicon phase gratings with periods of 2.4 µm over a beam size of 4.4 mm. The span in momentum space that was measured resulted in a reconstruction field of view that is the width of ten to twenty grating periods. While our technique is insensitive to a low density of grating defects, such as vacancies or dislocations, the overall shape and period of the grating is assumed to be uniform over the entire beam. If a PSD was used instead of the proportional counter used in this experiment, the periodic portions of the sample would only need to extend to the pixel size, placing the sub-micrometre imaging of quasi-periodic samples in reach. In practice, however, the per pixel count rate in such a setup would set an effective minimum beam size.

The phase-retrieval and tomographic reconstruction techniques demonstrated here may be useful for a sophisticated USANS spectrometer, such as those described in the works of Barker *et al.* (2005 ▸) or Strobl *et al.* (2007 ▸) for a wide variety of sample types. Phase retrieval and tomography is also used for X-ray coherent diffractive imaging (Shechtman *et al.*, 2015 ▸; Rodriguez *et al.*, 2013 ▸; Martin *et al.*, 2012 ▸; Burvall *et al.*, 2011 ▸; Langer *et al.*, 2008 ▸), and phase retrieval using the transport of intensity equation method for intermediate-field applications is used by phase-contrast neutron imaging (Allman *et al.*, 2000 ▸; Strobl *et al.*, 2009 ▸). The techniques described here are directly applicable to other far-field scattering measurements, such as small-angle neutron scattering (SANS), where the measured diffraction patterns are inherently two dimensional, instead of one dimensional, as is the case for USANS spectrometers.

## 2. Experiment   

**Figure 1:**
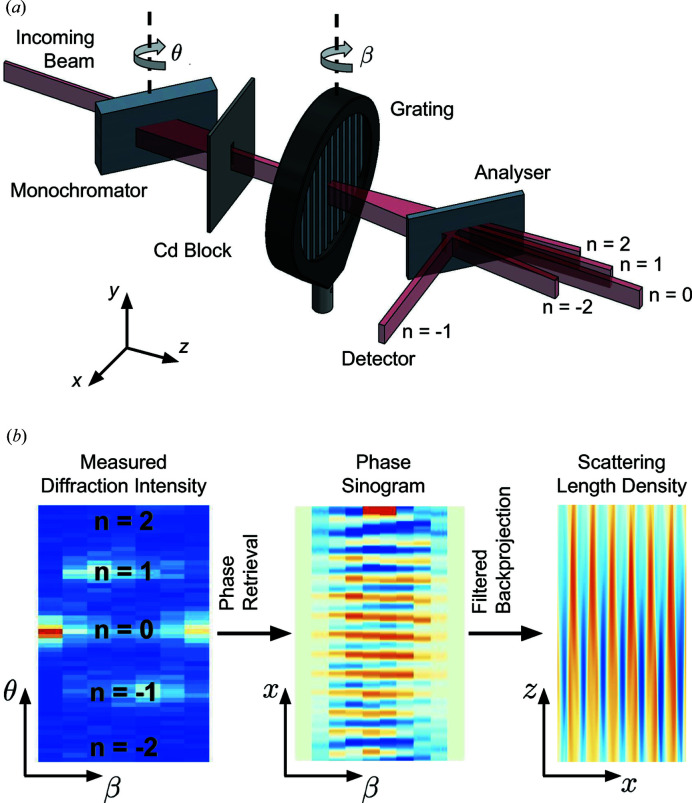
(*a*) The experimental setup. A λ = 4.4 Å neutron beam passes through a monochromator crystal, then through a phase grating whose effect is measured by an analyzer crystal and a ^3^He proportional counter. (*b*) From the measured diffraction intensity, the position-space phase is retrieved, providing the phase sinogram, which is then used to reconstruct the scattering-length density of the grating.

The experiment was performed at the NIOFa beamline at the National Institute of Standards and Technology (NIST) Center for Neutron Research (NCNR) in Gaithersburg, Maryland (Shahi *et al.*, 2016 ▸; Pushin *et al.*, 2015 ▸). A schematic of the experiment is shown in Fig. 1 ▸(*a*). A 4.4 Å wavelength neutron beam is extracted from the neutron guide using a pyrolytic graphite crystal. The beam passes through a 2 mm slit before being Bragg diffracted (Laue geometry) by a perfect-silicon crystal (111) monochromator. A 4.4 mm wide cadmium block is used to select the forward-diffracted beam from the monochromator. To measure the outgoing momentum distribution modified by the phase grating, we placed a second perfect-silicon crystal (111) analyzer after the grating, forming a double-crystal diffractometer. The monochromator was rotated relative to the analyzer by a rotation stage with an embedded angular encoder, allowing arcsecond-precision motion.

The Bragg-diffracted wavepackets are Lorentzian in shape in momentum space. Their nominal transverse coherence length is given by the *Pendellösung* length Δ_*H*_ = 35 µm for the 111 reflection from silicon at λ = 4.4 Å. Diffraction peaks with angular locations of
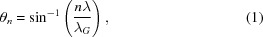
where λ_*G*_ is the period of the grating and *n* is an integer that represents the diffraction order, are clearly visible [Fig. 1 ▸(*b*)]. The relative amplitudes of the diffraction peaks depend on the shape and amplitude of the phase profile for a given angle of sample rotation β.

**Figure 2:**
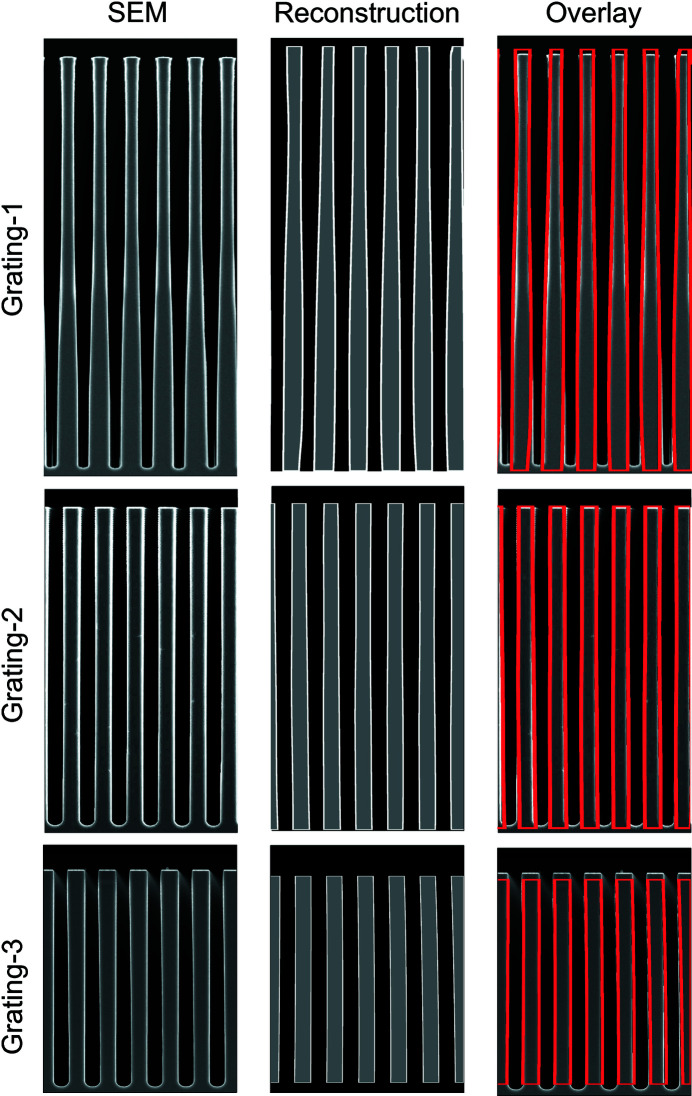
SEM micrographs of the phase gratings (left column) compared with their neutron tomographic reconstructions (middle column). Also shown is an overlay (right column) of the outline of the reconstruction over the SEM. The good agreement between the SEM micrographs and the reconstructions indicates that the shape of the gratings is uniform over a large range. The walls of Grating-2 and Grating-3 are shown to be very straight, while the sloped walls of Grating-1 appear in both the SEM micrograph and the reconstruction. Edge highlights are added to the reconstructions for clarity.

Three gratings were analyzed in this experiment. The period of each grating was λ_*G*_ = 2.4 µm. The grating depths were *h* = 29.0 µm, *h* = 23.9 µm and *h* = 15.8 µm, with corresponding phase amplitudes of 2.6 radians, 2.2 radians and 1.4 radians, respectively, for λ = 4.4 Å neutrons. Scanning electron microscope (SEM) micrographs of the three gratings are shown in Fig. 2 ▸.

Diffraction spectra as a function of grating rotation β about the *y* axis were taken from ∼−2 to 4° in 1° steps for Grating-1, −6 to 6° in 1° steps for Grating-2 and −6 to 6° in 1.5° steps for Grating-3. The −3° *y*-axis rotation diffraction spectrum for Grating-1 was substituted with the 5° spectrum because the −3° spectrum was out of the measured range. The grating rotation was severe enough for there to be no diffraction peaks expected in the −3° spectrum.

The position-space phase of each measured diffraction spectrum was computed using a phase-retrieval algorithm. The retrieved phase as a function of β forms a sinogram that was then tomographically reconstructed, providing two-dimensional images of the scattering-length density of the gratings [Fig. 1 ▸(*b*)]. See Section 3 for a detailed description of the reconstruction algorithm. The reconstructions of the grating scattering-length density and the SEM micrographs of the three gratings are shown in Fig. 2 ▸. The vertical walls of Grating-2 and Grating-3 are captured by the reconstructions, while the slope and curvature of the walls of Grating-1 visible in the SEM micrograph are well represented in the reconstruction. The good agreement between the SEM micrographs and the reconstructions also implies that the grating profile is uniform over a much larger region than what is visible to the SEM. A uniform phase profile is critical for neutron moiré interferometers, the recent demonstrations of which used the same phase gratings (Pushin *et al.*, 2017 ▸; Sarenac *et al.*, 2018 ▸).

The spatial resolution of the reconstructions is a fraction of the 2.4 µm period. We estimate the spatial resolution to be about one fourth of λ_*G*_/*n*_max_. The diffraction spectra were measured over a large-enough range to resolve *n* = 2 (which was highly suppressed for these phase gratings), so the ultimate resolution is estimated to be 300 nm.

## 3. Reconstruction algorithm   

The phase profile ϕ(*x*) of a neutron propagating in the *z* direction through a material is given in the Eikonal approximation (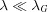) in the work of Sears (1989 ▸),
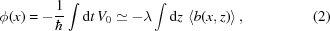
where λ is the neutron wavelength, *V*_0_ is the material’s optical potential, the integral is taken over the neutron’s trajectory, 〈*b*(*x*, *z*)〉 is the spatially dependent bound coherent scattering-length density and 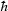 is the reduced Planck’s constant. For example, in a homogeneous material 〈*b*(*x*, *z*)〉 = *Nb*_c_ inside the material and 〈*b*(*x*, *z*)〉 = 0 outside the material, where *N* is the atomic number density and *b*_c_ is the bound coherent scattering length. The goal of tomography is then to reconstruct 〈*b*(*x*, *z*)〉. We consider one-dimensional phase profiles, though the extension to two dimensions is straightforward, in which case the reconstruction of the scattering-length density is three dimensional instead of two dimensional.

A sample that imprints a spatially dependent phase ϕ(*x*) over the incoming wavefunction Ψ_i_(*x*) results in an outgoing wavefunction of
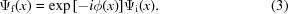
To analyze sample diffraction we look at the neutron wavefunction in momentum space:
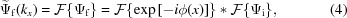
where 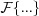 is a Fourier transform, and * is the convolution operator. In this experiment, we measure the outgoing neutron-momentum distribution, or diffraction spectrum, which is calculated as 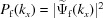.

If diffraction spectra are taken as a function of sample rotation, then the resulting momentum-space wavefunction is given by
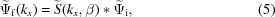
with the function 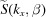 defined in terms of the the grating rotation angle β about the *y* axis (see Fig. 1 ▸) and
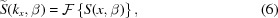
given that
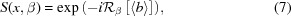
where integrating over the neutron’s trajectory through a sample as a function of sample rotation provides the phase profile [equation (2)] via a Radon transformation of the scattering-length density 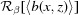. For clarity, we now drop the *k*_*x*_ and *x* arguments in 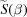 and *S*(β), respectively.

Because the size of the beam is much larger than the grating period, when *P*_f_(*k*_*x*_) is averaged over translations *x*_0_ of the incoming state, 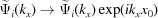, the measured momentum distribution reduces to
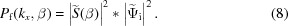
This reduction can also be viewed as an averaging over the phase, or physical translation in the *x* direction, of the periodic structure contained in ϕ(*x*). Thus, the result is independent of the translation of the periodic structure, rendering this technique insensitive to many types of sample defects.

Before recovering the phase profile ϕ(*x*), the measured momentum distribution is deconvolved with 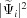, which is simply the measured diffraction spectrum with no sample present. For this experiment, discrete deconvolutions are performed between the measured momentum distributions for each *y*-axis rotation and the average of the first and last momentum distributions (largest grating rotations), where there were no visible diffraction peaks. Additionally, a two-dimensional Gaussian filter is applied to the resulting 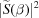 with respect to diffraction angle θ and grating rotation β for noise suppression. While this step is sufficient for our purposes, there are other methods for modifying phase-recovery algorithms for noisy data (Langer *et al.*, 2008 ▸; Martin *et al.*, 2012 ▸; Rodriguez *et al.*, 2013 ▸).

**Figure 3:**
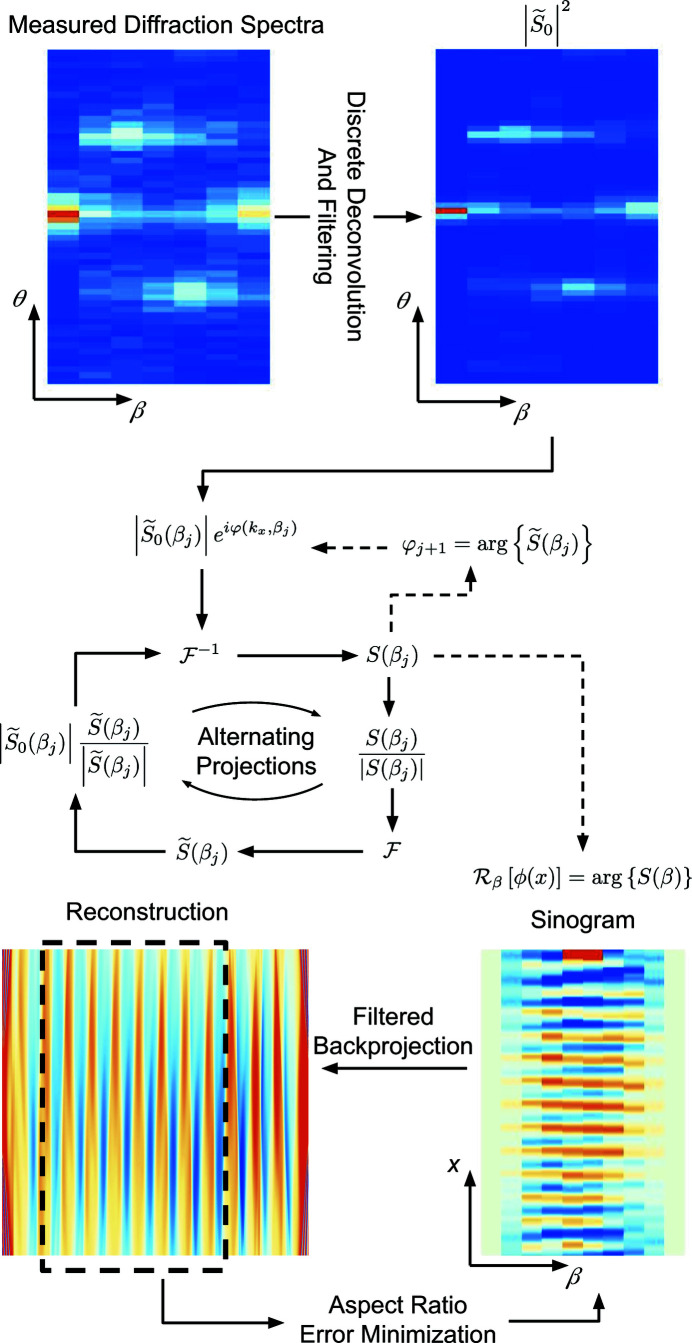
The outline of the entire reconstruction process. The raw data are filtered and deconvolved from the average diffraction spectrum of the maximum and minimum measured sample-rotation angles. The output is passed into an alternating projections algorithm for each sample rotation angle β, with the previous solution seeding the next step in β. A filtered back projection of the sinogram creates the reconstructed scattering-length density. The high-fidelity portion of the reconstruction is made into a binary image, Radon transformed back into a sinogram and compared with the original sinogram over the relevant range. The sinogram error is minimized with respect to the aspect ratio of the reconstruction. See text for details.

After isolating the deconvolved diffraction spectra 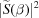, we compute
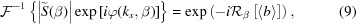
where φ(*k*_*x*_, β) is an unknown function. While the absolute value of 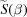 is known from the deconvolution of the incoming and outgoing momentum distributions, the phase φ(*k*_*x*_, β) has not been measured. Both the momentum-space phase φ(*k*_*x*_, β) and the position-space phase 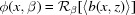 can be retrieved with an alternating projections algorithm (Shechtman *et al.*, 2015 ▸), an outline of which is shown in Fig. 3 ▸. Note that other methods of neutron phase recovery have also been demonstrated (Haan *et al.*, 2007 ▸).

### 3.1. Phase recovery   

Phase recovery by alternating projections works by alternating between real-space and Fourier-space magnitude constraints (Shechtman *et al.*, 2015 ▸). In our case, the Fourier-space magnitude-constraint step is imposed by updating 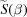 according to
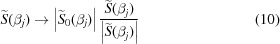
in Fig. 3 ▸, where *j* indexes the grating rotation angle and |*S*_0_(β_*j*_)| is the deconvolved measured diffraction spectrum. The real-space magnitude constraint comes from assuming that absorption is negligible, in which case |*S*(β)|^2^ = 1. The function *S*(β) is updated with each iteration of the algorithm by taking
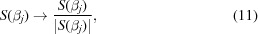
as shown in Fig. 3 ▸. Note that there are methods for extending the real-space constraint or phase recovery in general to samples with non-negligible absorption (Shechtman *et al.*, 2015 ▸; Burvall *et al.*, 2011 ▸).

It is well known that alternating projection algorithms can be sensitive to the initial phase guess φ(*k*_*x*_, β) as many global minima are possible (Shechtman *et al.*, 2015 ▸). Some of these minima may be physical, while others are not. In general, solutions are impervious to complex conjugations, phase offsets and real-space translations of *S*(β) (Guizar-Sicairos & Fienup, 2012 ▸). This complicates phase retrieval for the purposes of tomography because the solution space needs to be continuous between rotation steps, β_*j*_ → β_*j*±1_. A number of phase-retrieval algorithms for tomography exist (Burvall *et al.*, 2011 ▸; Langer *et al.*, 2008 ▸), but we find that a suitable way to ensure a continuous solution space as a function of grating rotation is to initiate the next iteration of β_*j*±1_ with the previous solution of φ(*k*_*x*_, β_*j*_) by taking
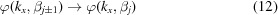
for an initial guess at each step in β. In our algorithm, we do this from the middle out, choosing the initial value of β to correspond to the grating approximately perpendicular to the beam, then seeding subsequent φ(*k*_*x*_, β) with that of the previous β in both the positive and negative sample-rotation directions.

The initial 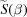 is found by passing a random phase φ(*k*_*x*_, β) into the alternating projections algorithm. This is repeated 200 times and the resulting solution with a minimum error in 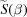 and *S*(β) is selected. The error is defined as
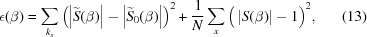
where *N* is the number of points in the *x* dimension of the array representing |*S*(β)|. This term gives equal weighting to the error in momentum space and position space because the discrete deconvolution process normalizes 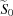, such that 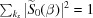. Finally, the retrieved phase of *S*(β) is identified as the sinogram of the sample’s scattering-length density 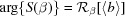.

### 3.2. Tomographic reconstruction   

The tomographic reconstructions of the recovered position-space phase are completed with a filtered back projection (Strobl *et al.*, 2009 ▸). In our case, we use a Hann filter, though other filtering functions may be selected. The aspect ratio of the reconstructed image is found by performing the filtered back projection, forming a binary image, then Radon transforming the resulting image and computing the error when compared with the original recovered phase sinogram. The error is minimized with respect to the aspect ratio of the reconstruction. The binarization of the reconstruction is completed by setting all the reconstruction values below the average to zero and all the values above the average to one. The minimization is a local minimum in the neighborhood of the aspect ratio as predicted by the known scattering-length density of silicon, the grating period measured using the diffraction-peak positions and equation (1), and the amplitude of the recovered phase sinusoid when the grating is approximately aligned with the beam. A comparison of the reconstructions and the SEM micrographs are shown in Fig. 2 ▸. The grating color was set to gray with the white outline added to make the images easier to compare. Note that other methods for discrete tomography (Krimmel *et al.*, 2005 ▸) may also be useful for samples made of a single material and cut to a certain shape.

### 3.3. Diffraction-spectrum truncation   

**Figure 4:**
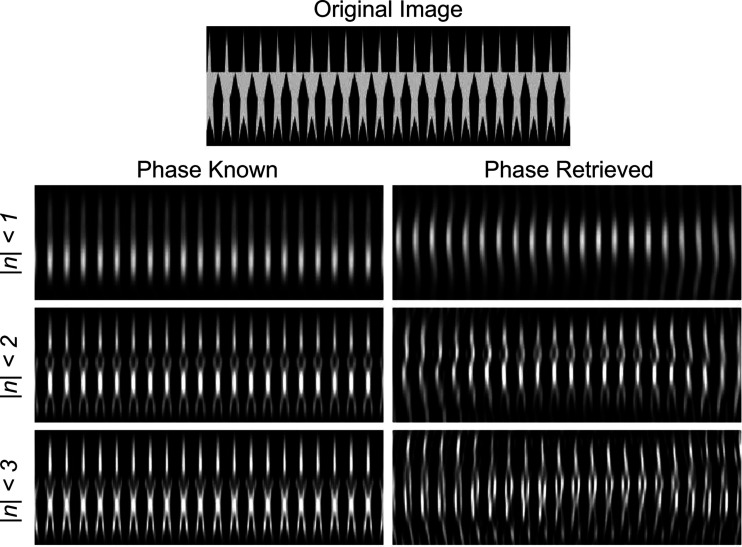
Results of the phase-retrieval algorithm and tomographic reconstruction with the FFT of a Radon-transformed image as inputs. A comparison of reconstructions after truncating 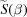 past *n*th order (rows) with the phase of 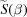 left intact (left column) and with the phase retrieved (right column).

High-order diffraction peaks are difficult to measure, both because their amplitudes tend to die off with increasing *n* and because spanning large momentum transfer requires longer measurement times. However, the resolution of the reconstruction will follow 2π/*Q*_tot_, where *Q*_tot_ is the total range in wavenumber transfer probed. Thus, there is a cost benefit to measuring higher diffraction orders versus taking more sample-rotation steps. However, tomographic reconstructions of the retrieved phase may also improve the spatial resolution of the reconstruction. This is because lower diffraction orders in the measured diffraction spectra 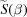 are a mixture of higher-order components of the Fourier decomposition of the underlying phase profile. For example, the *n* = 1 diffraction peak in 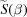 is a mixture of the *n* = 1 and the product of the *n* = 2 and *n* = −1 phase-profile Fourier coefficients, corresponding to the first-order and second-order term in the Born series for exp[−*i*ϕ(*x*)], respectively. In the limit of a single sample-rotation angle, CT is ill posed; however, one can still estimate a grating shape using a single recovered phase profile, as has been demonstrated with neutron diffraction at up to seventh order (Haan *et al.*, 2007 ▸). However, this method breaks down if the grating outline cannot be expressed as a single-valued function of the transverse coordinate. For example, the overhangs of the stars’ points in Fig. 4 ▸ can only be reproduced by measuring diffraction for multiple sample-rotation angles. Additionally, taking more projections at lower diffraction orders has a much lower requisite signal-to-noise ratio, whereas Haan *et al.* (2007 ▸) needed to resolve diffraction peaks that were four orders of magnitude less intense than the first-order peaks. The optimal choice in whether larger diffraction orders or a higher density of sample rotations should be pursued will probably depend on the overall phase shift of the sample. If samples with large phase shifts are measured, more information is contained in the amplitudes of the low diffraction orders as a function of sample rotation, as more terms in the Born series are relevant. However, the samples considered here, both virtual and experimental, have overall amplitudes of less than 2π.

To study the performance of the tomographic phase-retrieval algorithm as well as how truncating 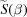 affects the reconstructions, we simulated diffraction spectra from the image shown in Fig. 4 ▸. Diffraction spectra were obtained from phase profiles of the Radon-transformed image from −19 to 19° in 2° steps. The diffraction spectra were then set to zero after the first-, second- or third-order diffraction peaks. The real-space phase was then reconstructed with (1) the phase of the truncated 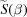 left intact and (2) after passing 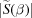 through the described phase-retrieval algorithm.

The reconstructions of the original image are shown in Fig. 4 ▸. The spatial resolution of the reconstructed images increases with increasing |*n*|_max_. The reconstructions where the phase was retrieved provide good estimations of the reconstructions using the known phase. However, some artefacts are evident for the phase-retrieved case. There tends to be a region of high fidelity, with distortions worsening further away horizontally. The region that best estimates the original image is not necessarily centered nor at the edge of the reconstruction because, as previously discussed in Section 3.1, phase-retrieval algorithms produce translationally invariant solutions. The distortions are of similar character for each level of diffraction-order truncation. Similar effects were seen in the reconstructions of the neutron phase gratings. The best region of the reconstruction was selected visually, as shown with the dashed line in Fig. 3 ▸.

## 4. Conclusions   

We find that the neutron diffraction from silicon phase gratings as a function of grating rotation can be used to tomographically reconstruct the shape of the gratings. These reconstructions rely on the periodic structure of the gratings but nonetheless have a spatial resolution of ∼300 nm, which is more than an order of magnitude smaller than other forms of neutron tomography. In principle, even smaller structures may be probed, in which case the spatial resolution of the reconstruction is nominally ∼2π/*Q*_tot_, with 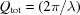 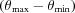, given by the rotational range of the double-crystal diffractometer. However, neutron-scattering-length densities for most materials are such that tens of micrometres of material are ordinarily required for a 2π phase amplitude given typical neutron wavelengths. Thus, it may be difficult to measure structures with amplitudes less than 100 nanometres.

An upper limit on the length scale to which this type of tomography is sensitive is set by the neutron coherence length of ∼35 µm. However, the addition of a PSD and possible combination of phase recovery with the USANS refractive signal (Treimer *et al.*, 2003 ▸) would allow for much larger reconstructions. Combining phase recovery with other tomography signals is intended for future work.

Further optimization of the phase-retrieval and reconstruction algorithms is probably possible. For example, selection of the width of the Gaussian filter employed in the deconvolution step is related to the noise present in the measurement of the diffraction spectra. Analysis of both real and simulated noisy data sets may elucidate how to best set this parameter. Optimization of the cost benefit between measuring larger diffraction angles versus taking a higher density of scans through β space is also of interest. This problem is probably dependent on the overall phase amplitude of the sample. One may study how these and other changes to the algorithm impact the reconstruction of digital phantoms when the fast Fourier transform (FFT) power spectra of the Radon-transformed image are used as inputs for the reconstruction, similar to Fig. 4 ▸. Finally, if the grating depth exceeds *h*_lim_ = λ_*G*_/σ_div_ ≃ 70 µm, where σ_div_ ≃ 0.5° is the beam divergence, the conical shape of the beam would need to be taken into account. Fortunately, there are pre-existing tomography algorithms for conical beams which could be utilized (Feldkamp *et al.*, 1984 ▸). However, such algorithms may lose their effectiveness if the grating depth is a few times larger than *h*_lim_. Despite the need for further improvements to the tomographic phase-retrieval algorithm, the results presented here indicate that high-quality tomographic reconstructions with sub-micrometre resolution of the scattering-length density are possible.

In the case of phase gratings, creating SEM micrographs entails cleaving the gratings, while our technique is both non-destructive and samples a much larger area of the gratings. Our method can provide non-destructive process and/or batch testing for nanofabrication applications. In cases where transporting nanofabricated samples to a neutron facility may not be practical or when the sample type does not benefit from using neutrons, phase-recovered tomography from far-field diffraction data could probably be extended to low-brilliance laboratory-scale X-ray sources.

In the same way that neutron CT from other signals can be used to visualize materials in a way that is complementary to X-rays (Strobl *et al.*, 2009 ▸), phase-retrieved neutron tomography can provide unique information about a sample. For example, a periodic structure buried in a matrix would produce a signal for neutrons but may appear opaque to other forms of radiation, which could be especially beneficial for probing tissue scaffolds such as those described in the work of Dvir *et al.* (2011 ▸). Lithium ion batteries can be imaged using neutrons (Siegel *et al.*, 2011 ▸), and batteries with electrode layers that are too thin for traditional neutron imaging, such as those described in the work of Zhang *et al.* (2011 ▸), could benefit from this method. Finally, we plan to adapt our algorithm to accommodate samples made of two or more materials, in which case it could potentially be of use for holographic studies of colloids, where treating colloid structures as a perturbation to the scattering signal from a grating has been shown to be inadequate for neutron studies (Feng *et al.*, 2018 ▸).

Two-dimensional phase retrieval and three-dimensional tomography could be achieved if data are taken as a function of more than one sample axis of rotation. For a USANS setup, this would probably be a combination of *y*-axis and *z*-axis rotation in Fig. 1 ▸, since only diffraction along the *x* axis is measured. For SANS data, where the diffraction spectra are inherently two dimensional, only one axis of rotation is required for three-dimensional tomography. If the algorithm is adapted to SANS data, in addition to measuring the spacing of oriented biological membranes (Nagy *et al.*, 2011 ▸), the shape of the membranes could be reconstructed. The technique could also easily be extended to polarized neutron beams to study magnetic samples. For example, the depth profile of magnetic vortices could be probed (Eskildsen *et al.*, 2009 ▸; Kawano-Furukawa *et al.*, 2011 ▸; Butch, 2018 ▸). Additionally, given the utility of measuring skyrmion lattices using SANS (Mühlbauer *et al.*, 2009 ▸; Adams *et al.*, 2011 ▸), it may be possible to generate three-dimensional renderings of skyrmions and provide important insights into their shape (Gilbert, 2018 ▸). Finally, any samples with periodic structures that have a macroscopic ordering and exhibit diffraction peaks in SANS or USANS data are candidates for phase-recovered neutron tomography. For an unpolarized test case, we have successfully analyzed the shape of neutron phase-grating walls, confirming the tomographic reconstructions with SEM micrographs.
